# OnPLS integration of transcriptomic, proteomic and metabolomic data shows multi-level oxidative stress responses in the cambium of transgenic hipI- superoxide dismutase Populus plants

**DOI:** 10.1186/1471-2164-14-893

**Published:** 2013-12-17

**Authors:** Vaibhav Srivastava, Ogonna Obudulu, Joakim Bygdell, Tommy Löfstedt, Patrik Rydén, Robert Nilsson, Maria Ahnlund, Annika Johansson, Pär Jonsson, Eva Freyhult, Johanna Qvarnström, Jan Karlsson, Michael Melzer, Thomas Moritz, Johan Trygg, Torgeir R Hvidsten, Gunnar Wingsle

**Affiliations:** 1 Umeå Plant Science Centre, Department of Forest Genetics and Plant Physiology, Swedish University of Agricultural Sciences, SE-90183 Umeå, Sweden; 2 Umeå Plant Science Centre, Department of Plant Physiology, Umeå University, SE-90187 Umeå, Sweden; 3 Department of Chemistry, Umeå University, SE-90187 Umeå, Sweden; 4 Computational life science cluster (CLiC), Department of Chemistry, Umeå University, Umeå, Sweden; 5 Department of Mathematics and Mathematical Statistics, Umeå University, SE-90187 Umeå, Sweden; 6 Department of Clinical Microbiology, Clinical Bacteriology, Umeå University, SE-90187 Umeå, Sweden; 7 Department of Molecular Cell Biology, Institute of Plant Genetics and Crop Plant Research, 06466 Gatersleben Germany; 8 Department of Chemistry, Biotechnology and Food Science, Norwegian, University of Life Sciences, 1432 Ås Norwegian, Norway; 9 Division of Glycoscience, School of Biotechnology, Royal Institute of Technology, AlbaNova University Centre, S-106 91 Stockholm, Sweden; 10 Department of Civil, Environmental and Natural Resources Engineering, Sustainable Process Engineering, Luleå University of Technology, 971 87 Luleå, Sweden

**Keywords:** Statistical integration, OnPLS, Poplar, Oxidative stress, Systems biology

## Abstract

**Background:**

Reactive oxygen species (ROS) are involved in the regulation of diverse physiological processes in plants, including various biotic and abiotic stress responses. Thus, oxidative stress tolerance mechanisms in plants are complex, and diverse responses at multiple levels need to be characterized in order to understand them. Here we present system responses to oxidative stress in *Populus* by integrating data from analyses of the cambial region of wild-type controls and plants expressing high-isoelectric-point superoxide dismutase (hipI-SOD) transcripts in antisense orientation showing a higher production of superoxide. The cambium, a thin cell layer, generates cells that differentiate to form either phloem or xylem and is hypothesized to be a major reason for phenotypic perturbations in the transgenic plants. Data from multiple platforms including transcriptomics (microarray analysis), proteomics (UPLC/QTOF-MS), and metabolomics (GC-TOF/MS, UPLC/MS, and UHPLC-LTQ/MS) were integrated using the most recent development of orthogonal projections to latent structures called OnPLS. OnPLS is a symmetrical multi-block method that does not depend on the order of analysis when more than two blocks are analysed. Significantly affected genes, proteins and metabolites were then visualized in painted pathway diagrams.

**Results:**

The main categories that appear to be significantly influenced in the transgenic plants were pathways related to redox regulation, carbon metabolism and protein degradation, e.g. the glycolysis and pentose phosphate pathways (PPP). The results provide system-level information on ROS metabolism and responses to oxidative stress, and indicate that some initial responses to oxidative stress may share common pathways.

**Conclusion:**

The proposed data evaluation strategy shows an efficient way of compiling complex, multi-platform datasets to obtain significant biological information.

## Background

Comprehensive profiling of transcriptional regulation coupled with proteomic and metabolomic measurements would greatly facilitate characterization of changes in levels of important compounds during cellular regulation as e.g. oxidative stress [1]. However, few attempts have been made to extensively investigate cellular metabolism under stress conditions [2,3]. Furthermore, such studies have previously focused on acquiring and integrating data at only two omic levels (either transcriptomic and metabolomic, or transcriptomic and proteomic) [2,3]. Since any systems-level response is a result of complex interplay between gene regulation, post-translational modifications and metabolic fluxes, these studies might have missed responses visible only by investigating all three omics-levels simultaneously. The multi-omic profiling required for full analysis would generate a very large, complex dataset, and biologically meaningful interpretation of such datasets requires use of powerful systems biology techniques for integrating multidimensional information into networks [4]. Numerous strategies have been proposed for integrating data from parallel sources [3,5,6], and a multivariate regression method O2PLS, and its extension OnPLS, have been recently shown to be promising tools for integrating multi-omic plant data [7-10].

In plants, reactive oxygen species (ROS) are involved in diverse physiological and developmental processes [11,12]. However, various abiotic or biotic stressors may disrupt the cellular redox state, thereby causing levels of ROS to rise [13] and inducing a range of protective mechanisms that promote the recovery of redox balance and recuperation from the toxic effects of excess ROS [14]. ROS can be viewed as signals produced in real time for the fine tuning of plant developmental and metabolic processes; and redox regulation may occur under different growth conditions and with diurnal variations [15]. Depending on the inductive conditions, oxidative stress may also induce programmed cell death (PCD) in plants [11], but several reports indicate that different concentrations of ROS are required for inducing PCD than those causing non-specific cellular damage [1,16]. Thus, redox metabolism and responses are complex, and known to be controlled by an intricate regulatory network of which many aspects are poorly understood [1].

In order to study oxidative stress responses, we have used wild-type (WT) controls and transgenic hybrid aspen plants expressing a high-isoelectric-point superoxide dismutase (hipI-SOD) gene in antisense orientation [17]. HipI-SOD is a Cu/Zn-SOD with a suggested role in ROS regulation and plant development [18-20]. The transgenic hipI-SOD *Populus* plants have higher levels of O_2_^-^ than WT counterparts and impaired growth rates, accompanied by histological and morphological perturbations, including compressed and disorganized cell structures in the cambial region of the stem Srivastava et al. [17] (Additional file 1: Figure S1). This region is also one of the sites of both suppression of the hipI-SOD protein, according to immunolocalization analysis (Srivastava et al. [18]), and increased O_2_^-^ production in the transgenics. The cambium generates cells that differentiate to form either phloem or xylem. Hence the oxidative stress caused by overproduction of O_2_^-^ in the cambial region of transgenics is hypothesized to be a major reason for their phenotypic perturbations. Thus, we postulated that the region would be an ideal model system to study the effects of oxidative stress on plant development *in vivo*.

**Figure 1:**
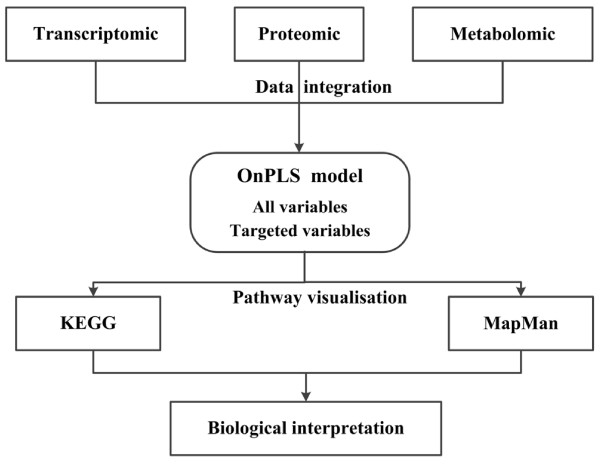
**Schematic flowchart of the integrated profiling strategy applied in this study.** In the first step, transcriptomic, proteomic and metabolomic data were collected individually from the cambial region of *Populus* WT and transgenic plants. In the second step, the three omic datasets acquired were integrated by OnPLS to identify the joint variation in them (initially applying OnPLS modeling to all variables, and subsequently to targeted variables). Finally, the OnPLS model from the second step was visualized by Mapman and KEGG to explore the pathways (genes-proteins-metabolites) affected in the transgenics, and deepen the interpretation of their oxidative stress responses.

In the presented study we applied a systems biology approach to analyze effects of oxidative stress in *Populus*. We first acquired transcriptomic, proteomic and metabolomic profiles of the cambial region of two different transgenic hipI-SOD lines and WT control hybrid aspen plants and then applied the multivariate analysis method OnPLS to integrate the three levels of omics data. One OnPLS model was built from all genes, proteins and metabolites (i.e. all variables), and one model was built using only identified compounds (targeted variables). OnPLS modeling facilitates the detection of connections in datasets that are intrinsically linked by flows of information (e.g. transcript-protein-metabolite flows). This is obtained through the interpretation of joint scores and loadings, prediction of the globally joint variation and correlated biological interpretation of the datasets. OnPLS reduces the error that might arise in the process of investigating several model diagnostics and latent variables to see which different combination such as transcript-protein, transcript-metabolite, protein-metabolite should be joined first. The OnPLS approach does not depend on the order in which the matrices are processed when one have more than two blocks of data, and thus the model is symmetrical giving no preference to any matrix [9,10]. Finally, we used the genes, proteins and metabolites identified as significantly affecting the transgenic aspens to identify affected pathways and examined them according to the measured abundances of genes, proteins and metabolites in transgenic and control plants. Here, the results are summarized, and the biological pathways are interpreted in the context of existing knowledge to extend understanding of system-level responses to oxidative stress in plants. The information acquisition, analysis, visualization and interpretation steps in the study are schematically illustrated in the flowchart shown in Figure 1.

## Methods

### Plant materials

Samples of the cambial region were obtained at the same time of the day from three 12-week-old WT plants, and from three plants of each of two antisense lines (AS-SOD9 and AS-SOD24) [17,18]. After peeling away the bark from each plant, tissue from the cambial region (5–18 internodes) was scraped from the bark side with a scalpel frozen in liquid nitrogen as described by Celedon et al. [21]. All samples were ground in a mixer-mill (MM 301, Retsch GmbH, Germany) and the resulting tissue powder was used for analysis or kept at −80°C until further use.

### Experimental design

For microarray experiments, mRNA samples from each of the nine plants were hybridized against a combined sample pool of mRNA (with equal contributions from each of the plants) in a dye-swap design. In total, 18 arrays were hybridized. In both the proteomic and metabolomic experiments, each of the nine samples was analyzed three times.

### Transcriptome analysis

cDNA clones and mRNA samples were prepared, labeled and hybridized for transcript profiling using POP2.3 cDNA microarrays as previously described by Bylesjö et al. [8] with a few modification. Briefly, total RNA was extracted from 30 mg of tissue powder using an Aurum total RNA mini kit (Bio-Rad) according to the manufacturer’s instructions. Approximately 1 μg of total RNA was used to selectively amplify mRNA using a MessageAmp™ II aRNA Amplification Kit (Ambion, Cat. AM1751). 10 μg of amplified RNA (a-RNA) was reverse-transcribed into aminoallyl-labeled cDNA with 3 μg of Random Primer Nanomer. All slides were scanned four times with predefined laser power (50–100) and phototube multiplier (PMT; 70–80) settings using a ScanArray 4000 (Perkin-Elmer Wellesley, MA, USA). The resulting images were analyzed in GenePix Pro 5.1 (Molecular Devices, CA, USA), and the extracted data were stored as results files containing raw data and various statistical measurements. All original image files and raw data are available online for download from the UPSC-BASE microarray database [22] (http://www.upscbase.db.umu.se) under experiment UMA-0080. The different scan levels for the slides were merged with Restricted Linear Scaling (RLS) [23] followed by step-wise normalization as previously described by Wilson et al. [24]. Flagged spots were treated as missing values and normalized intensities below 7 were set to 7 in a censoring procedure as previously described by Ryden et al. [23] to reduce the influence of non-expressed genes. Values obtained from each plant’s two dye-swap replicates were combined into a single gene-expression vector (ignoring missing values). From the 27,963 probe spots, 14,619 genes were obtained after filtering according to the procedure of Sterky et al. [25]. Lists of significantly differentially expressed genes and common names of genes discussed in the manuscript can be found in Additional file 2: Table S1.2.

### Proteome analysis

Proteins were extracted from 20 mg of frozen tissue powder from each plant as described by Bylesjö et al. [8]. After extraction, proteins were reduced by adding DTT solution to a final concentration of 15 mM and incubated at 55°C for 45 min. All samples were then alkylated by adding iodoacetamide solution (final concentration, 80 mM) and incubating them for 30 min at room temperature (RT) in the dark. The extracted proteins were subsequently placed in pre-wetted Microcon filter tubes (Ultracel YM-10, Millipore, USA), centrifuged at 12 000 g for 15 min at RT and washed three times with 0.2 M ammonium bicarbonate. Approximately 0.6 μg of trypsin (Promega/SDS Biosciences) in 0.2 M ammonium bicarbonate was then added to each sample and they were digested overnight (~16 hrs) at 37°C. The resulting peptides were collected in a new collection tube by three repeated centrifugations with 50 μL of 0.2 M ammonium bicarbonate, dried and redissolved in 0.1% formic acid for peptide analysis by reversed-phase liquid chromatography electrospray ionization mass spectrometer (LC-ESI-MS), as described by Bylesjö et al. [8], using a nanoACQUITY ultra-performance liquid chromatography (UPLC) system coupled to a Q-TOF mass spectrometer (Q-TOF Ultima; Waters Corp.). Each sample was loaded onto a C18 trap column, (Symmetry 180 μm × 20 mm 5 μm; Waters, Milford, MA) and washed with 2% acetonitrile, 0.1% formic acid at 15 μL/min for 2 min. The samples were eluted from the trap column and separated on a C18 analytical column (75 μm × 100 mm 1.7 μm; Waters, Milford, MA) at 400 nL/min using 0.1% formic acid as solvent A and 0.1% formic acid in acetonitrile as solvent B, in a gradient. The following gradients were used: linear from 0 to 40% B in 25 min, linear from 40 to 80% B in 1 min, isocratic at 80% B in 1 min, linear from 80 to 5% B in 1 min and isocratic at 5% B for 7 min. The eluting peptides were sprayed into the mass spectrometer with the capillary voltage set to 2.1 kV and cone voltage to 40 V. MS spectra were collected in the 400–1300 m/z range (0.8 s scan time, 0.1 s inter delay). Instrument and offset calibration was performed as described by Srivastava et al. [18] with a randomized run order of samples to minimize the influence of systematic time drift.

### Protein identification

Three sample mixtures were created by separately pooling all WT and both transgenic (AS-SOD9 and AS-SOD24) peptide samples. Each sample mixture was then analyzed nine times at different predefined mass ranges (400–500, 500–600, 600–650, 650–700,700-750, 750–800, 800–900, 900–1000 and 1000–1300 m/z) by using the same chromatographic gradient as described above. Peptide fragmentation data were generated by automated Data Dependent Acquisition (DDA) and submitted for database searches (*Populus* protein database; 45 555 entries, assembly release version 1.1) using previously described settings from Bylesjö et al. [8], except that peptide tolerance was set to 100 ppm and fragment tolerance to 0.1 Da. Proteins were classified as identified if at least two peptides (where one peptide was sequence unique) with a Mascot score exceeding the statistically relevant threshold (p < 0.05) were found, or just one unique peptide with the required Mascot score was found, that yielded at least four consecutive y- or b-ions with significant signal to background ratios. A total of 424 proteins were identified. A concatenated target-decoy database-search strategy was used to check the false discovery rate (FDR), which was found to be less than 1.5%. Data for unique peptides with an e-value < 0.1 were exported in xml format for quantification.

### Peptide quantification

The MS raw data files were converted to mzXML files using massWolf (version 4.3.1). The MS mzXML and MASCOT xml files were parsed and processed with a program developed in-house. Briefly, each scan was subjected to smoothing using Savitzky-Golay [26] filtering (second order polynomial, five data points, two iterations) and peak areas were calculated after noise reduction. Peak mass was set to the average of the three highest data points for each peak.

Unique peptides identified with MASCOT were matched to the parsed MS data using the parameters detected m/z, charge state and retention time, using a retention time window of ± 1.0 min. Charge states were calculated by using the first three isotopic peaks of a peptide and the same mass tolerances for detecting the mono isotopic peak as in the MASCOT search. In order to minimize the number of false positive hits, only peaks with at least three identifiable isotopic peaks showing a correct isotopic pattern were accepted as matches, i.e. for peptides with a mass less than 1800 Da, when measured as M + H, the mono isotopic peak had to be the highest and the third isotopic peak the lowest, with no peak of significant intensity at a m/z below that of the mono isotopic peak within the m/z range corresponding to the charge state of the peptide. The chromatographic peak shape was determined by identifying a peptide in subsequent scans of the MS channel and the area under the curve was calculated by summing the intensities for the first three isotopic peaks for each peptide over its chromatographic peak. A total 458 unique peptides, corresponding to 271 proteins were quantified and used in the OnPLS analysis for all variables, out of which 243 were used in the targeted analysis. Significantly differently expressed proteins and common names of proteins discussed in the manuscript are listed in Additional file 2: Table S2.2. The data are deposited in the PRIDE database (accession numbers 31652 to 31654; http://www.ebi.ac.uk/pride/).

### Metabolome analysis

Gas chromatography–mass spectrometry (GC-MS) and liquid chromatography-mass spectroscopy (LC-MS) were used for the metabolomic analysis, as follows.

### GC-MS analysis

Metabolites were extracted, and their profiles in all samples were analyzed by GC-MS as described by Bylesjö et al. [8] with no modifications.

### UPLC-MS analysis

Chromatography was performed using a Waters Acquity UPLC system, equipped with column oven, coupled to a Micromass LCT Premier time-of-flight (TOF) mass spectrometer equipped with an electrospray source operating in negative/positive ion mode in W mode with lockspray interface for accurate mass measurements. The source temperature was 120°C with a cone gas flow of 10 L/hr, a desolvation temperature of 320°C and a nebulizing gas flow of 600 L/hr. The capillary voltage was set at 2.5 kV for negative ion mode and at 3.0 kV for positive ion mode, with a cone voltage of 35 V, a data acquisition rate of 0.15 s, and interscan delay of 0.1 s, with dynamic range enhancement (DRE) mode activated. Leucine enkephalin was employed as the lockmass compound, infused straight into the MS at a concentration of 500 pg/μL (in 50:50 acetonitrile:water) at a flow rate of 30 μL/min. The normal lockmass in the DRE mode was the C13 peak of leucine enkephalin at 555.2645 in negative ion mode and the C13 peak at 557.2800 in positive ion mode; the extended lockmass peak was the normal ion peak observed at 554.2615 in negative ion mode and at 556.2771 in positive ion mode. All mass spectral data were acquired in the centroid mode, 100–1000 m/z, with a data threshold value set to 2.

A 2 μL aliquot of extracted sample (4°C) was injected onto a 2.1 × 100 mm, 1.7 μm BEH C18 UPLC column (Waters) held at 40°C in a column oven. The gradient elution buffers were A (H_2_O, 0.1% formic acid) and B (acetonitrile, 0.1% formic acid), and the flow-rate was 500 μl min^-1^. The column was eluted with the following gradient: 1-20% B over 4 min, 20%-40% B over 2 min, 40%-95% B over 3 min, then 4.5 min isocratic 95% B. The UPLC-ESI/MS instrumentation was operated by the MassLynx™ v4.1 software (Waters, UK), and the acquired data was processed by the QuanLynx™ software (Waters, UK).

### Structural identification with UHPLC-LTQ/Orbitrap mass spectrometry

For structural elucidation of the phenolic compounds, high mass accuracy MS and tandem mass spectrometry (MSMS) analysis were performed using an LTQ/Orbitrap mass spectrometer (Thermo Fisher Scientific, Bremen, Germany) with an ESI source. Chromatographic separation was performed with a Thermo Accela LC system, with a column oven (held at 40°C). The eluents, column and mobile phase gradient were the same as for the UHPLC-ESI-TOF-MS. Profile mass spectra were collected in the Orbitrap mass analyzer, operating in negative ionization mode, with a target mass resolution of 30 000 (full width at half maximum peak height, defined at m/z 400). Indicated MS/MS spectra were collected after collision-induced dissociation (CID) in the LTQ cell, using normalized collision energy of 35%. External mass calibration was performed according to the manufacturer’s guidelines. Elemental composition of ions was calculated from the accurate masses with Xcalibur QualBrowser software (Thermo Scientific).

### Metabolite identification GC-MS and LC-MS

GC-MS detected peaks were identified by comparing their mass spectra and chromatographic retention indices with those of entries in Umeå Plant Science Center’s in-house MS library or the mass spectra library of the Max Planck Institute in Golm (http://csbdb.mpimp-golm.mpg.de/csbdb/gmd/gmd.html), using NIST MS-Search version 2.0 (NIST, Gaithersburg, MD). A total of 350 putative metabolites (all variables) were detected in the analysis, of which 56 were identified (targeted variables). To identify peaks detected by LC-MS, their accurate masses, retention times and MS-MS spectra were solely compared to those of entries in the in-house library. From the LC-MS analysis in negative mode, a total of 4230 mass features (metabolites) were detected, of which 36 gave distinct fingerprints (all variables) and five were positively identified (targeted variables). Metabolites identified in both the GC-MS and LC-MS analyses are listed in Additional file 2: Table S3.1. The datasets for the metabolomics data (GC-MS and LC-MS) are available at the UPSC database (http://www.upsc.se/metabolomicsdata) with the experiment number GC20131010.

### Experiment workflow and data integration by OnPLS

The OnPLS method can handle noisy, multicollinear datasets with many more variables than observations (samples), which is a typical situation in biochemical and biological applications. Data acquired from all platforms were initially preprocessed, prior to integration by OnPLS. The transcript datasets were log2-transformed and mean-centered per microarray. The transcriptomic, proteomic and metabolite (extracted chromatographic peak) data for the transgenics were all normalized, relative to WT, by scaling each value to unit variance with the mean and standard deviation of the corresponding WT data. The WT values were used as internal references across the profiling platforms [8].

OnPLS [9,10] is a recently published extension of O2PLS [27,28] that generalizes to multiblock cases where several blocks of data are subjected to analysis. The problem that O2PLS aims to solve is described as follows: Given two data blocks *X*_1_ (*M* × *N*) and *X*_*2*_ (*M* × *K*) we can split the variation in each ***X*** block into two parts, *X* = *X*_*J*_ + *X*_*U*_ *+ E,* where ***X***_*J*_ and ***X***_*U*_ correspond to the joint and unique variation respectively. E is the residual matrix. The joint variation is overlapping and shared between the data blocks and the unique variation is present only in that data block. O2PLS was developed for two blocks of data and OnPLS is a recent generalization for more than two blocks of data providing symmetry in the modeling and thereby enhancing the interpretation; compared to O2PLS where models are obtained from an order dependent analysis.

**Figure 2:**
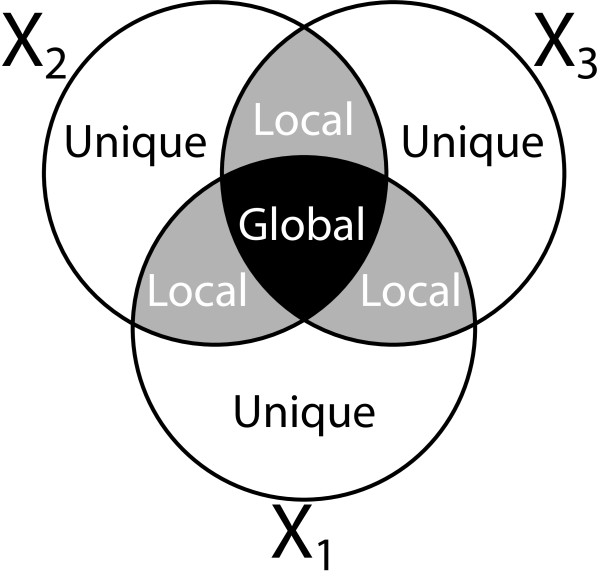
**An illustration of what OnPLS does for three blocks*X***_***1***_***X***_***2***_**and *X***_**3.**_ It separates each block into the parts that it has in common with the other blocks. The parts are globally joint (shared between all blocks), locally joint (shared between some, but not all, blocks) and unique, shared with no other block.

OnPLS [9,10] models the *globally joint* variation (shared between all blocks), the *locally joint* variation (variation that is shared between some, but not all blocks) and the *unique* variation (variation in one block not shared with any other block). A graphical overview of this is presented in Figure 2.

As an example, the first matrix in an OnPLS model for three blocks obtains the decomposition

$$\begin{array}{l} X_{1}=\underset{\mathrm{globally}\;\mathrm{joint}\;\mathrm{part}}{\underset{︸}{\left(X_{1}\cap X_{2}\cap X_{3}\right)}}+\underset{\;\mathrm{locally}\;\mathrm{joints}\;\mathrm{parts}}{\underset{︸}{\left(\left(X_{1}\cap X_{2}\right)\backslash X_{3}\right)+\left(\left(X_{1}\cap X_{3}\right)\backslash X_{2}\right)}}\\ \;+\underset{\mathrm{unique}\;\mathrm{part}\;\mathrm{part}}{\underset{︸}{\left(X_{1}\cap \bar{\left(X_{2}\cup X_{3}\right)}\right)}} \end{array}$$

where ∪ is the set union operator, ∩ is the set intersection operator, ∖ is the set difference operator and $\bar{X}$ is the set complement. See Reference [29] for a detailed description of theory and method of OnPLS.

The variable importance values (VIP) [30] were used to select the most important variables that were also significant according to the Jack-knifed confidence interval (Zamboni et al. [31], Bylesjö et al. [7,8]). Variables having VIP values exceeding 0.5 were deemed to be significant.

### Pathway analysis

Efficient visualization tools are required for robust systems biological interpretation of the high-dimensional data generated from combined profiling (transcriptomic, proteomic and metabolomic) [32]. For this purpose we used two freely available software packages: Paintomics Version 2.0 (http://www.paintomics.org; Garcia-Alcalde et al. [33]) to map and visualize the gene, protein and metabolite measurements in KEGG pathways; and MapMan (http://mapman.gabipd.org; Thimm et al. [34]) to visualize the transcriptomic and proteomic variables, as well as transcripts/proteins not described by KEGG. These packages provide efficient tools for visualizing metabolic differences between the transgenic and WT plants, and characterizing the key affected molecular processes. All targeted variables in the transcriptomics, proteomics and metabolomics datasets with their identified pathways are listed in Additional file 2: Table S1.1, Additional file 2: Table S1.2, Additional file 2: Table S2.1, Additional file 2: Table S2.2 and Additional file 2: Table S3.1, Additional file 2: Table S3.2, respectively. Subcellular localization of the proteins was derived from the Arabidopsis Information Resource (TAIR; http://arabidopsis.org) and listed in Additional file 2: Table S4.1.

## Results and discussion

Contrary to our previous work which was focused on the whole stem and apical parts of the plants (Srivastava et al. 2007) [17], the present study focused on the specific cambium region to explore the role of ROS on plant development. Similar studies in plants have focused more on single gene, protein and metabolite responses, selected pathway or transcript-protein, transcript-metabolite or protein-metabolite interactions; however this study is focused on all levels [35-43]. The global approach presented here is needed in order to effectively target and elucidate multi-level oxidative response in plants [44,45].

### Integrated omics data (transcript, protein and metabolite levels) by OnPLS

**Figure 3:**
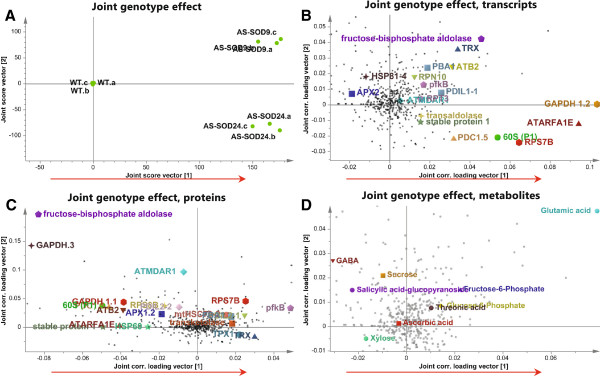
**The genotype effect. (A)** Joint genotype effect scores from the targeted variable model, and corresponding loadings for: **(B)** transcripts, **(C)** proteins and **(D)** metabolites. Many significant compounds are highlighted in their respective plots and discussed in the text. The directions of the “red colored” arrow heads and tails represent increasingly high levels of transcripts/proteins/metabolites in the transgenic hipI-SOD plants relative to WT plants, and vice versa, respectively. The variation in the WT is too small to be visible on this score plot.

We built two OnPLS models, one based on all variables (genes, proteins and metabolites) and one based only on the targeted variables (genes with corresponding proteins, and identified metabolites). In this way, we used the first model for exploratory purposes and the second model for biological interpretation including the investigation of relationships between gene and protein expression [46]. The first model was built based on 14,619 genes, 271 proteins and 386 metabolites (350 GCMS and 36 LCMS). This OnPLS model had two globally joint components between all three blocks capturing 70% of the variation in transcripts, 96% in proteins and 86% in metabolites. The second, targeted OnPLS model was built based on 243 transcripts and proteins (proteins were matched to their corresponding gene names) and 61 identified metabolites. This OnPLS model had two globally joint components capturing 89% of the variation in transcripts, 96% in proteins, and 95% in metabolites. The coefficient of variations for each genotype was 0.1% (WT), 5% (AS-SOD9) and 2% (AS-SOD24), which shows that the biological variability is small compared to the variation linked to the mutation (between groups) as observed visually in the score plot (Figure 3a). This is expected given the fundamental effect the transformation had on the metabolism.

Overall the targeted dataset go in the same direction as the dataset containing all variables, and so we focused our analysis on the globally joint components of the targeted model. From the targeted model, 65 (Additional file 2: Table S1.2) out of 243 genes (Additional file 2: Table S1.1), 85 (Additional file 2: Table S2.2) out of 243 proteins (Additional file 2: Table S2.1) and 29 (Additional file 2: Table S3.2) out of 61 identified metabolites (Additional file 2: Table S3.1) were significantly affected in the transgenics. The targeted OnPLS model revealed that the joint covariance captures genotype effects, which distinctly separate the transgenic plants from WT counterparts (Figure 3a). However, a clear difference between the two transgenic lines was also observed. Figure 3 shows the results of the integrated analysis. All four plots in Figure 3 are connected through the joint variation. The joint genotype effect observed in the transcripts, proteins and metabolites respectively are displayed in separate plots to facilitate the interpretation. Aldolase, glyceraldehyde-3-phosphate dehydrogenase (GAPDH), 4-aminobutyrate (GABA) and other variables highlighted in Figure 3b,c,d are discussed in the text.

Tables 1 and 2 provide arrows indicating up-regulation or down-regulation for the significantly differentially expressed proteins, transcripts and metabolites, respectively. Additional information of these is also found in Additional file 2: Table S1.2; Additional file 2: Table S2.2; Additional file 2: Table S3.2. Although several loci encoding for proteins may have the same activity, paralogs of protein have been shown to have different functions (e.g. due to duplication of the genome as in poplar) [47], therefore quantitative data on protein level and their corresponding transcript have been selected for comparison in this targeted approach (Table 1). As the data of the two transgenic lines are normalized with respect to WT, only two sources of variation exist in the data; how the transgenic lines differ from WT and how they differ between themselves. In the following sections we will only focus on how the transgenic lines differ from WT using the targeted OnPLS model.

**Table 1:** Overview of protein and transcript that are significantly differentially expressed in the targeted OnPLS model

**JGI V2.2 ID**	**MapMan name**	**MapMan symbol**	**AS-SOD9**			**AS-SOD24**		
			**P**	**/**	**T**	**P**	**/**	**T**
POPTR_0002s14740	**Amino acid metabo.**	3-phosphoshikimate 1-carboxyvinyltransferase	↑	/	↑	↑	/	↑
POPTR_0013s05850		ATCIMS (cobalamin-independent methionine synthase)	↑	/	―	↓	/	―
POPTR_0010s16420	**Cell wall**	RGP3 (reversibly glycosylated polypeptide 3)	↓	/	↓	↓	/	↑
POPTR_0017s13350		RGP2 (reversibly glycosylated polypeptide 2)	↓	/	↓	↓	/	↓
POPTR_0017s12760		UDP-glucose 6-dehydrogenase	↑	/	―	↓	/	―
POPTR_0009s13270	**Cell.cycle**	Peptidyl-prolyl cis-trans isomerase/cyclophilin (CYP2)/rotamase	↓	/	―	↓	/	―
POPTR_0003s21080	**Cell.organisation**	TUA5 (tubulin alpha-5)	↓	/	―	↓	/	―
POPTR_0002s09610		ANNAT1 (annexin arabidopsis 1)	↓	/	―	↓	/	―
POPTR_0001s31700		ACT7 (actin 7)	↓	/	―	↓	/	―
POPTR_0001s04180		TUA5 (tubulin alpha-5)	↓	/	―	↓	/	―
POPTR_0001s29670		TUA6 (tubulin alpha-6 chiain)	↓	/	―	↓	/	―
POPTR_0002s11250		TUA6 (tubulin alpha-6 chiain)	↓	/	―	↓	/	―
POPTR_0006s15090	**DNA.synthesis**	3, NFA3	↑	/	↓	↓	/	↓
POPTR_0009s03310		H2B/HTB2 (histone h2b)	↓	/	―	↓	/	―
POPTR_0016s12760	**Fermentation**	Pyruvate decarboxylase	↓	/	↑	↓	/	↑
POPTR_0001s38560	**Gluconeogenesis**	Malate dehydrogenase (NAD), mitochondrial	↑	/	―	↓	/	―
POPTR_0006s17940	**Glycolysis**	Fructose-bisphosphate aldolase	↑	/	↑	↓	/	↑
POPTR_0015s14380		LOS2 (low expression of osmotically responsive genes 1)	↑	/	―	↓	/	―
POPTR_0010s06560		GAPDH.3 (glyceraldehyde-3-phosphate dehydrogenase (GAPDH))	↓	/	―	↓	/	―
POPTR_0012s09570		GAPDH 1.1	↓	/	―	↓	/	―
POPTR_0006s11400		2,3-biphosphoglycerate-independent phosphoglycerate mutase	↑	/	―	↓	/	―
POPTR_0002s23510	**Hormone metabolism**	ATB2	↓	/	↑	↓	/	↑
POPTR_0001s05100	**Lipid metabolism.**	MOD1 (mosaic death 1)	↓	/	―	↓	/	―
POPTR_0007s01850	**Major CHO metabolism**	pfkB-type carbohydrate kinase family protein	↑	/	↑	↑	/	↑
POPTR_0002s14730		Transketolase	↑	/	―	↑	/	―
POPTR_0001s05690		AAC2 (ADP/ATP carrier 2)	↑	/	―	↓	/	―
POPTR_0002s25950	**Misc.**	Acid phosphatase class B family protein	↓	/	―	↓	/	―
POPTR_0006s18240		GDSL-motif lipase/hydrolase family protein	↑	/	―	↓	/	―
POPTR_0010s15250		Tropinone reductase, putative/tropine dehydrogenase	↓	/	↓	↓	/	↑
POPTR_0007s07960	**N-metabolism**	ATGSR1 (Arabidopsis thaliana glutamine synthase clone R1)	↑	/	―	↓	/	―
POPTR_0013s05480		GDH1 (glutamate dehydrogenase 1)	↓	/	―	↓	/	―
POPTR_0001s10670	**Nucleotide metabolism**	NDPK1 (nucleoside diphosphate kinase 1)	↑	/	―	↓	/	―
POPTR_0001s32490	**Protein.aa activation**	Methionine--tRNA ligase, putative/methionyl-tRNA synthetase	↑	/	―	↓	/	―
POPTR_0006s24090	**Protein.degradation**	APM1 (aminopeptidase M1)	↓	/	―	↓	/	―
POPTR_0016s12720		ATG2 (G2p-related protein)	↓	/	―	↓	/	―
POPTR_0005s02520		RPT5A (regulatory particle triple-A 5A)	↓	/	―	↓	/	―
POPTR_0018s14290		PBA1 (20S proteasome beta subunit A 1)	↑	/	↑	↑	/	↑
POPTR_0009s15910	**Protein.folding**	Chaperonin	↓	/	―	↓	/	―
POPTR_0001s35790	**Protein.folding**	Chaperonin	↓	/	―	↓	/	―
POPTR_0001s14040		HSP60 (Heat shock protein 60)	↓	/	―	↓	/	―
POPTR_0008s04230	**Protein.synthesis**	Elongation factor 1-alpha/EF-1-alpha	↑	/	↑	↓	/	↑
POPTR_0001s23190		Elongation factor 1-beta/EF-1-beta	↑	/	―	↓	/	―
POPTR_0012s09840		Elongation factor 1-beta, putative/EF-1-beta	↓	/	↓	↓	/	↓
POPTR_0002s05220			↑	/	―	↓	/	―
POPTR_0009s12150		40S ribosomal protein S25 (RPS25B)	↑	/	―	↑	/	―
POPTR_0016s05530		40S ribosomal protein S2 (RPS2C)	↓	/	―	↓	/	―
POPTR_0008s04400		40S ribosomal protein S23 (RPS23B)	↓	/	―	↓	/	―
POPTR_0006s21210		ATRPS5B (ribosomal protein 5B)	↓	/	―	↓	/	―
POPTR_0004s09830		40S ribosomal protein S25 (RPS25B)	↑	/	↑	↑	/	↑
POPTR_0001s26950		40S ribosomal protein S8 (RPS8B)	↑	/	―	↓	/	―
POPTR_0002s24410		60S ribosomal protein L13A (RPL13aC)	↓	/	―	↓	/	―
POPTR_0002s14250		60S ribosomal protein L15 (RPL15A)	↓	/	―	↓	/	―
POPTR_0012s03450		60S ribosomal protein L19 (RPL19B)	↓	/	―	↓	/	―
POPTR_0004s07620		60S ribosomal protein L19 (RPL19B)	↓	/	―	↓	/	―
POPTR_0001s35630		60S ribosomal protein L27 (RPL27C)	↓	/	―	↓	/	―
POPTR_0008s05970		60S ribosomal protein L35a (RPL35aC)	↓	/	―	↓	/	―
POPTR_0018s13700		60S ribosomal protein L7 (RPL7C)	↓	/	―	↓	/	―
POPTR_0002s18010		60s acidic ribosomal protein P1	↓	/	↑	↓	/	↑
POPTR_0013s01220	**Protein.targeting**	AT-IMP (Arabidopsis thaliana importin alpha)	↓	/	―	↓	/	―
POPTR_0002s19210		ATARFA1E (ADP-ribosylation factor A1E)	↓	/	↑	↓	/	↑
POPTR_0008s12550	**PS.lightreaction.**	ATP synthase beta chain 2, mitochondrial	↓	/	↓	↓	/	↑
POPTR_0006s11570	**Redox.**	ATMDAR1 (monodehydroascorbate reductase 1)	↑	/	―	↓	/	―
POPTR_0006s13440		APX2 (ascorbate peroxidase 2)	↓	/	―	↓	/	―
POPTR_0001s44990		TPX1 (thioredoxin-dependent peroxidase 1)	↑	/	―	↑	/	―
POPTR_0003s11350		ATHIP1 ( HSP70-interacting protein 1)	↓	/	―	↓	/	―
POPTR_0005s25420		ATTRX1 (Arabidopsis thaliana thioredoxin H-type 1)	↑	/	↑	↑	/	↑
POPTR_0002s19940		ATPDIL2-1/MEE30/UNE5 (PDI-like 2–1)	↑	/	―	↑	/	―
POPTR_0014s15820		ATPDIL2-2 (PDI-like 2–2)	↑	/	―	↓	/	―
POPTR_0018s03000	**RNA**	Chloroplast nucleoid DNA-binding protein	↓	/	―	↓	/	―
POPTR_0004s16260		ATGRP7 (cold, circadian rhythm, and rna binding 2)	↑	/	↑	↑	/	↑
POPTR_0002s03580	**Secondary metabolism**	Isoflavone reductase	↓	/	―	↓	/	―
POPTR_0002s10000	**Signaling**	GRF2 (general regulatory factor 2)	↓	/	―	↓	/	―
POPTR_0004s17840	**Stress.abiotic.cold**	ATGRP2B (glycine-rich protein 2B)	↑	/	↓	↓	/	↓
POPTR_0001s18040		HSP91 (heat shock protein 91)	↓	/	―	↓	/	―
POPTR_0009s08320		mtHSC70-2 (heat shock protein 70)	↑	/	―	↑	/	―
POPTR_0004s04450		Pollen Ole e 1 allergen and extensin family protein	↓	/	―	↓	/	―
POPTR_0008s16670	**TCA**	Malate dehydrogenase, cytosolic	↓	/	―	↓	/	―
POPTR_0004s07320	**TCA**	Isocitrate dehydrogenase	↑	/	―	↓	/	―
POPTR_0013s11070	**Transport.misc**	SEC14 cytosolic factor family protein	↓	/	↓	↓	/	↓
POPTR_0001s36710	**Not assigned**	2-oxoacid dehydrogenase family protein	↑	/	↑	↓	/	↑
POPTR_0010s16050		Stable protein 1-related	↓	/	↑	↓	/	↑
POPTR_0602s00200		VEP1 (vein patterning 1)	↓	/	―	↓	/	―
POPTR_0017s10720		Unknown protein	↓	/	―	↓	/	―
POPTR_0017s13710		Unknown protein	↑	/	―	↑	/	―
POPTR_0005s26930		Unknown protein	↑	/	―	↑	/	―

Columns indicate *Populus trichocarpa* gene ID; Mapman name with similar classes grouped in rows; Mapman symbol; and AS-SOD9, AS-SOD24 protein (P) and transcript (T) expression levels, respectively. Symbols indicate: ↑, upregulated (relative to WT); ↓, downregulated; - not significantly differentiated expression. Data shown in Additional file 2: Table S2.2, Additional file 2: Table S1.2.

**Table 2:** Overview of metabolites (GC-MS and LC-MS) that are significantly differentially expressed in the targeted OnPLS model

**Class**	**Metabolite**	**AS-SOD9**		**AS-SOD24**
**Phenolic glycoside**	Cinnamoyl-hexose	↑	/	↑
	Coumaroyl-hexose	↑	/	↑
	Ferulate-glycoside	↑	/	↑
**Amino Acid**	3-Cyanoalanine	↑	/	↑
**Amine alcohol**	Ethanolamine	↓	/	↓
**Amino acid**	Glutamic acid	↑	/	↑
	Ornithine	↑	/	↑
	Arginine	↑	/	↑
	GABA (4-aminobutyric acid)	↓	/	↓
	Aspartic acid	↑	/	↑
	Cycloleucine	↑	/	↑
	Pyroglutamic acid	↑	/	↑
	Phenylalanine	↑	/	↓
	Valine	↑	/	↑
	Glycine	↑	/	↑
**Dicarboxylic acid**	Glutaric acid	↑	/	↑
**Disaccharide**	Disaccharide	↑	/	↑
	Sucrose	↑	/	↓
**Flavonoid**	Flavonoid	↑	/	↑
**Glucopyranoside**	Salicylic acid-Glucopyranoside	↓	/	↓
**Hexose phosphate**	Fructose-6-Phosphate	↑	/	↑
	Glucose-6-Phosphate	↑	/	↑
**Hydroxy acid**	Shikimic acid	↑	/	↑
**Nucleoside**	Uridine	↓	/	↓
**Organic acid**	Threonic acid	↑	/	↑
	Oxalic acid	↑	/	↑
**Phosphate**	Inositol phosphate-like	↓	/	↑
**Trisaccharide**	Raffinose	↑	/	↓
	Xylose	↓	/	↓

Columns indicate: metabolites with similar classes grouped in rows; metabolite name; and abundance in AS-SOD9 and AS-SOD24 (relative to WT). Symbols indicate: ↑, upregulated; ↓, downregulated. Data shown in Additional file 2: Table S3.2.

The majority of the significant regulated transcripts and metabolites from the OnPLS targeted analysis showed an up-regulation in the transgenic lines (Tables 1 and 2, Additional file 2: Table S1.2; S3.2). However, the proteins showed an opposite tendency, where most of the proteins were down-regulated (Table 1). The direction (up or down-regulation) of the response to the stress between the transgenic lines, AS-SOD9 and AS-SOD24, was relatively similar for the significant transcripts, proteins and metabolites (approximately 80% of the variance, Tables 1 and 2, Additional file 2: Table S1.2; Additional file 2: S2.2; Additional file 2: S3.2). When comparing the coregulation between the protein and the corresponding transcript, extracted from Table 1, it was found to be low (13%).

Steady state levels of the proteome depend on transcription, the levels of the transcripts, translation and protein degradation. Here we find diverse examples of regulation when we compare protein and transcript levels during perturbation by superoxide in the cambial region of *Populus*. Several studies have found a poor link between changes in transcript and protein levels in response to perturbation [48-50]. The regulation of changes in mRNA level is predominately regulated at the level of transcription while mRNA degradation is generally constant in mammalian cells [51]. For proteins levels it has been found that protein synthesis rates are the primary drivers of differentiation [52]. However, these authors conclude that transcriptomes and proteomes correlate very poorly because there is still substantial variance imparted at the level of protein synthesis and degradation. Another suggested concept was that if protein expression can be analysed, they could be used to formulate a more accurate biological predictions than what mRNA expression changes alone would yield [53]. The strength in our experiment however is the integration of transcripts, proteins and metabolites, to obtain significant biological information.

### Biological interpretation

**Figure 4:**
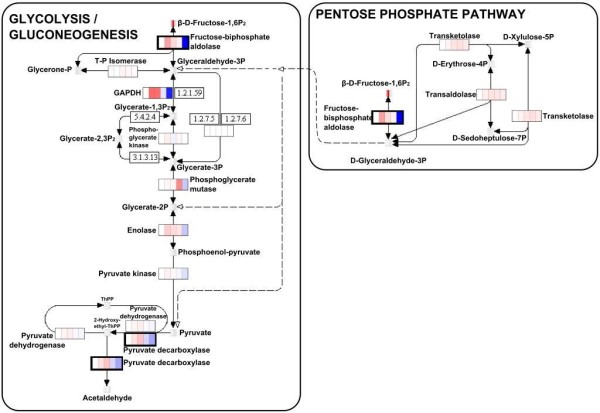
**KEGG Glycolysis/Gluconeogenesis pathway and Pentose Phosphate Pathways ‘painted’ with transcriptomic, proteomic and metabolomic data from the targeted OnPLS model**. Black-bordered entry boxes indicate significant differences between the transgenic and WT plants at both transcript and protein levels. The first three sections of each gene box (left to right) indicate WT, AS-SOD9 and AS-SOD24 transcript levels, respectively, and the last two protein levels in AS-SOD9 and AS-SOD24 lines, respectively. The first sections in the metabolite entry boxes represent WT and the colored boxes levels in the AS-SOD9 and AS-SOD24 lines. Reduced levels in the transgenics are colored blue and increased levels red.

After ‘painting’ KEGG and MapMan pathway maps with the omics datasets, we found several interesting pathways associated with differential transcripts, proteins and metabolites (Additional file 2: Table S1.2, Additional file 2: Table S2.2, Additional file 2: Table S3.2). Figure 4 shows sections of the KEGG Glycolysis/Gluconeogenesis and Pentose Phosphate Pathway maps, some features of which are discussed below.

In the transgenic trees, high expression levels were detected for proteins related to ROS detoxification and maintenance of cells’ redox balance. Cytosolic ascorbate peroxidase (APX2) protein (POPTR_0006s13440.1) and transcript (POPTR_0016s08580.1) levels were lower (relative to WT), and monodehydroascorbate reductase (MDAR1, POPTR_0006s11570.1) protein levels were higher in AS-SOD9 and lower in AS-SOD24 plants, which may be indicative of prolonged, severe oxidative stress. Moreover, there was a pronounced accumulation of threonate, a breakdown product of ascorbate, in both transgenic lines. Ascorbate is one of the principal antioxidant molecules in the cell and the production of ascorbate breakdown products indicates a failure to recycle all of the oxidized ascorbate via the ascorbate-glutathione cycle [54,55]. The observed expression levels of APX2 (cytosolic) and MDAR1 (peroxisomal) might be influenced by their localization in different compartments and linked to ascorbate and threonine levels. APXs have been reported to have declining activity with sensitivity to low ascorbate concentration [56,57] and induction on mRNA level [58].

A cytosolic thioredoxin (TRX) h-type1 paralog (POPTR_0005s25420.1) was induced at both protein and transcript levels in the transgenic lines. In addition, a thioredoxin-dependent cytosolic peroxidase protein (TPX1, POPTR_0001s44990.1) was upregulated in the transgenic lines. TRXs are small, ubiquitous proteins involved in the reduction of disulfide bridges in a variety of target enzymes that are present in all sub-cellular compartments and involved in many biochemical reactions. Thus, they have major effects on the post-translational modification of proteins and redox homeostasis, since dithiol-disulfide exchange reactions are heavily involved in both of these processes. These types of proteins play important roles in protecting organisms against the toxic effects of ROS and regulating intracellular signal transduction [59,60].

Other proteins that are linked to stress and redox regulation and were differentially expressed in the transgenic lines, relative to WT, were heat shock proteins (HSPs), protein disulfide isomerase (PDI), glycine-rich RNA-binding proteins, actin and tubulins [61]. Elevated levels of the mtHSC70 protein (POPTR_0009s08320), was found in the transgenic lines. AtDjB1, in association with mtHSC70, functions as an ATPase and plays a crucial role in limiting oxidative damage caused by heat stress [62]. The protein level of paralogs of PDIL-2 (PDIlike-2, POPTR_0002s19940 and POPTR_0014s15820) increased in the transgenic lines without a corresponding increase in transcripts. PDI contains thioredoxin (TRX) domains and act as a catalyst of disulfide bond formation in the oxidizing environment of the ER, hence stabilizing the tertiary and quaternary structures of protein folding [63]. Interestingly, in *Arabidopsis* PDI2 was suggested to have functional roles in the nucleus, interacting with the nuclear embryo transcription factor MEE8, in addition to its more studied role in the ER lumen [64]. Another sign of increased oxidative stress in the transgenic lines is upregulated levels of flavonoid, which probably will have antioxidant capacity, in the transgenic lines [65].

We found that carbon metabolism pathways, such as the glycolysis/gluconeogenesis and pentose phosphate pathway (PPP) were strongly affected in the transgenics. Affected components of the glycolysis/gluconeogenesis KEGG pathway included pyruvate decarboxylase (POPTR_0016s12760.1, PDC1.5), which was upregulated at transcript level but downregulated at protein level (Figure 4), and a cytosolic fructokinase (POPTR_0007s01850.1), which was upregulated at both transcript and protein levels. Further indications of shifts in the transgenics’ carbon metabolism include the following: Fructose-bisphosphate aldolase (POPTR_0006s17940.1) and glyceraldehyde-3-phosphate dehydrogenase (POPTR_0015s10330.2, GAPDH 1.2) was upregulated at the transcript but downregulated at the protein level (POPTR_0010s06560.1 GAPDH.3, POPTR_0012s09570.1 GAPDH 1.1; Additional file 2: Table S1.2, Additional file 2: Table S2.2; Table 1). Transaldolase (POPTR_0003s16030.1) and transketolase (POPTR_0002s14730.1), both of which provide reversible links between the PPP and glycolysis [66], were upregulated at transcript and protein levels respectively. PPP and glycolysis have been suggested to contribute to ROS balance and scavenging [67-69]. The upregulation of the glycolysis participants fructose-6-phosphate and glucose-6-phosphate, in conjunction with an observed downregulation of sucrose, xylose and upregulation of transketolase (key components of the PPP), is indicative of a shift towards the breakdown of carbohydrates with a profound rearrangement of primary carbon metabolism in response to an imbalanced redox state in the transgenics. These findings suggest that there are strong connections between glycolysis, PPP, carbon metabolism and oxidative stress, possibly resulting in enhanced reducing power in the form of increased levels of NADPH or NADH, thus raising the capacity for reductive biosynthesis [69,70]. These observations support the hypothesis that the remodeling of carbon metabolism may be part of an “emergency strategy” that reroute the metabolic flux from glycolysis to the PPP as an immediate and protective response to counteract oxidative stress [70]. This hypothesis has to be validated in plants since most of the experiments supporting this have been performed in other systems and mainly on the transcript level [66,68]. Although several studies have discussed the glycolysis-PPP complex pathway relationship in metabolites and transcripts [71,72], there is a need for future detailed multi-level (transcript-protein-metabolite) study of these two pathways in plants.

One group of proteins that was highly downregulated in the transgenic plants was the ribosomal proteins (r-proteins; Additional file 2: Table S1.2, Additional file 2: Table S2.2; Table 1). However, transcripts encoding the r-proteins showed an opposite trend. Ribosome biogenesis and mRNA translation are highly energy-demanding processes. Thus, limitations in energy supply restrict translation capacity (as well as cell growth and differentiation). Low energy levels trigger cells to switch to an energy preservation mode, in which essential cell functions and viability are maintained, but ribosome biogenesis is inhibited. The downregulation of r-protein biogenesis in the transgenic plants discussed here supports the hypothesis that it might be part of a reprogramming of plant’s energy transformation and utilization machinery under energy limitations.

The 26S proteasome is highly abundant both in the nucleus and cytosol, controlling central cellular signaling processes. Mis-folded and otherwise defective proteins are eliminated by degradation, frequently by 26S proteasomes following ubiquitin-tagging [73,74]. In addition, free 20SP has been shown to be able to use oxidized proteins as targets in a Ub-independent pathway, i.e. it does not require a poly-(Ub)-tag for proteasomal degradation. Here, RPN10 (regulatory particle non-ATPase subunit 10, POPTR_0004s17940.1) and RPT3 (AAA-ATPase subunit, root phototropism 3, POPTR_0016s02790.1) was upregulated at the transcript level, but protein levels of these components were not affected in the transgenic plants. Furthermore, PBA1 (20S proteasome beta subunit A1, POPTR_0018s14290.1) was upregulated at both protein and transcript levels in the transgenic plants.

In the transgenic lines glutamate was highly upregulated and GABA downregulated. These two compounds participate in the GABA shunt, a metabolic pathway that bypasses two steps of the TCA cycle [75]. The major role of GABA in plants has been suggested to be in primary metabolism, but it may possibly also act as a signal [76]. We observed reduced levels of a salicylic-sugar conjugate, salicylic-glucopyranoside, in the transgenic plants, indicating that changes in salicylic acid metabolism that promote reductions in ROS levels may be involved in oxidative stress responses [77].

## Conclusion

The objective of the presented study was to obtain information about multi-level (transcriptomic, proteomic and metabolomics) responses to oxidative stress in a specific cell tissue, the cambium, in our model *Populus* system. Data integration was based on the OnPLS method for its unique features of handling complex multi-omic datasets, extracting global and locally joint variations from them and, thus, facilitating the acquisition of biological understanding. OnPLS provided information about functional and pathway responses to oxidative stress in the examined transgenic plants. Global correlation values were obtained, confirming the utility of the strategy and highlighting the need for further development and application of OnPLS-based methods in systems biology.

The biological results obtained of the global responses to oxidative stress indicated the following responses: First, as the plants were stressed, antioxidant processes were induced to cope with the oxidative stress, resulting in misfolding and a subsequent degradation of oxidized proteins that appeared to take place via an induced, free 20S proteosome. Secondly, the sugars needed for energy production to keep minimal processes were activated via glycolysis and PPP, highlighting a somewhat unknown role of PPP in oxidative stress in *Populus* model system and need for further proteomic validation in plants. Downregulation of protein synthesis was also observed, which should provide major savings in energy consumption. These responses indicate the induction of maturation and cell death-associated signals in the transgenics, in addition to defense responses. Thus, our results suggest that premature maturation events (e.g. cell death) also occur in response to prolonged abiotic stress. Furthermore the results illustrate a divergence in transcript and protein levels and thus demonstrate the requirement of combined analysis to make an adequate biological interpretation.

In summary, we have hypothesized a biological sequence of responses that we can envisage from our combined “omics” study. However, we do realize that further validation have to be performed. All platforms, transcriptomics, proteomics and metabolomics, develop rapidly and will help to gain more and better information in the imminent future. But we strongly believe that one important approach to gain knowledge in cell biology is to combine results from different types of analyses, as done and shown here with the OnPLS method.

## Competing interests

We declare that we do not have competing interests.

## Authors’ contributions

VS, OO, GW Conceived and designed the experiment, VS, OO, JB, TL, PR, RN, MA, AJ, PJ, EF, JQ, JK, MM, TM, JT, TRH, GW conducted the experiments and analyzed the data, VS, OO, JB, MA, TM, JT, TRH, GW drafted the manuscript, TM, JT, TRH, GW supervised the project. All authors have read and approved the final version of this manuscript.

## Supplementary Material

Additional file 1: Figure S1 Light micrographs of transverse sections of stems of WT, AS-SOD9 and AS-SOD24 plants (A-C, respectively) and electron micrographs showing ultrastructural features of their cambium cells (D-I). Click here for file

Additional file 2: Table S1.1 Genes encoding examined transcripts, their KEGG designations and relative abundance in WT and transgenic hipI-SOD plants. **Table S1.2.** Genes with significantly changed transcript abundance in transgenic hipI-SOD plants compared with WT sorted by KEGG pathways and MapMan designations. **Table S2.1.** List of unique proteins quantified and compared in transgenic hipI-SOD and WT plants. **Table S2.2.** Proteins with significantly different abundance in transgenic hipI-SOD plants compared with WT. **Table S3.1.** List of metabolites identified by mass spectrometry (GC-MS and LC-MS). **Table S3.2.** Metabolites with significantly different abundance in transgenic hipI-SOD plants compared with WT (detected by GC-MS and LC-MS). **Table S4.1**. Subcellular localization of proteins in transgenic hipI-SOD plants compared with WT sorted by location designation. Click here for file
