# Supplementary material for: OnPLS integration of transcriptomic, proteomic and metabolomic data shows multi-level oxidative stress responses in the cambium of transgenic hipI- superoxide dismutase Populus plants
Source: BMC Genomics. 2013 Dec 17;14:893. doi: 10.1186/1471-2164-14-893 (PMC3878592; doi:10.1186/1471-2164-14-893)
Supplement: Additional file 1: Figure S1 — Light micrographs of transverse sections of stems of WT, AS-SOD9 and AS-SOD24 plants (A-C, respectively) and electron micrographs showing ultrastructural features of their cambium cells (D-I). [file 1471-2164-14-893-S1.pdf]

Supplementary Figure S1:

Fixation, substitution and embedding for light and transmission electron microscopy

Preparation of 2mm thick stem cross sections for light microscopy and ultrustructural analysis was carried out as described by Tognetti et al.,(2006), except that an FEI Tecnai G2 Sphera transmission electron microscope (FEI, http://www.fei.com) was used for ultrastructural analysis at 120 kV.

Results

Comparison of the cambium region of cross-sections of the tenth internode of WT and transgenic plants showed histological differences (Figure S1). WT shows 10-12 rows of cambium cells which look well organized (Figure S1A), while antisense line AS-SOD24 showed reduced rows of cambium cells (Figure S1B). Antisense line AS-SOD9 is characterized by less cambium cells which appears compressed and disorganized (Figure S1C). On an ultra-structural level cambium cell of WT did not show plasmolysis and the cytoplasm appears denser (Figure S1D, G) and show more cell organelles compared to line AS-SOD24 which shows slightly plasmolysis and looser cytoplasm (Figure S1E, H). Cambium cells of line AS-SOD9 is characterized by plasmolysis and the cytoplasm appears reduced (Figure S1F, I).

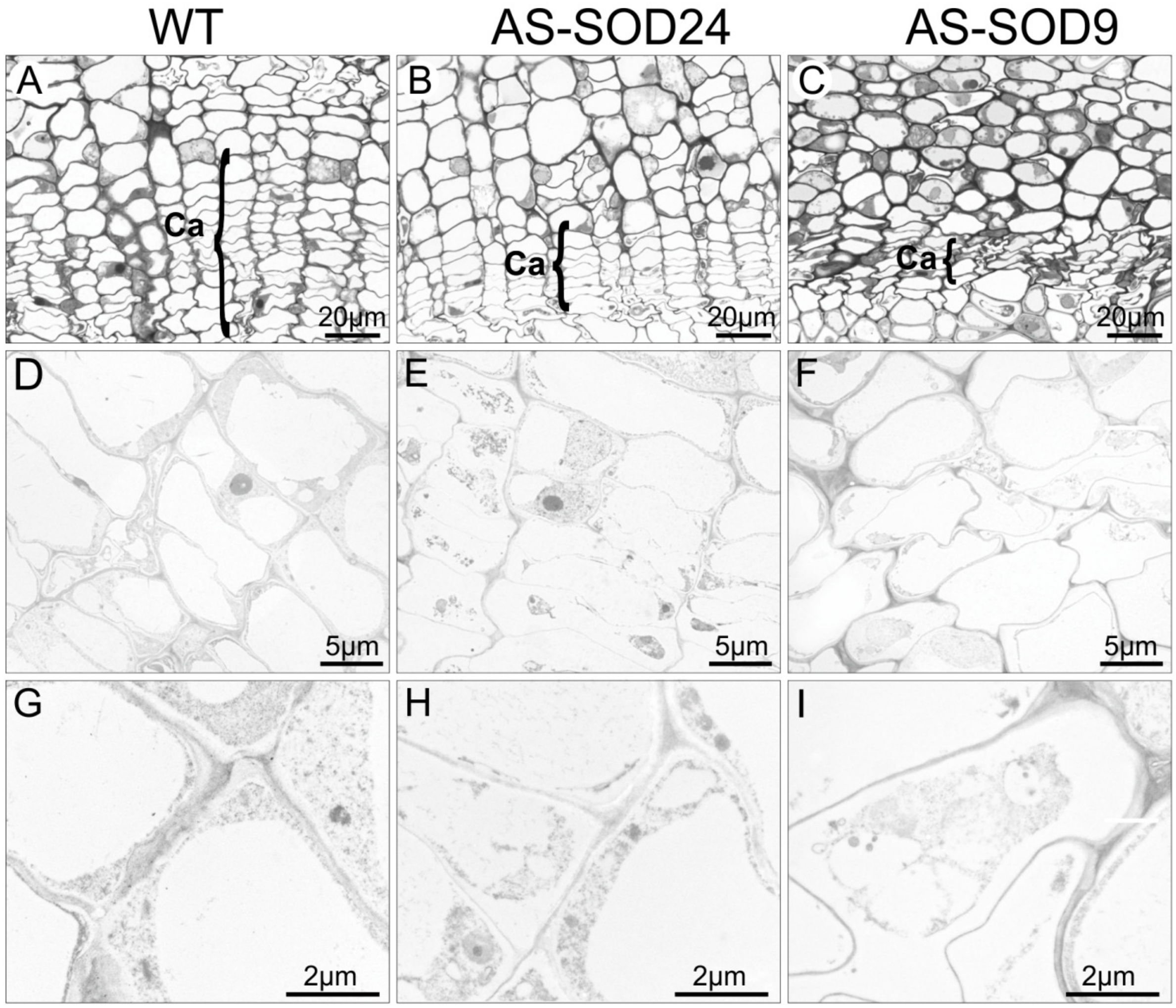

Supplemental Figure S1: Light micrographs of transverse sections of stems of WT, AS-SOD9 and AS-SOD24 plants (A-C, respectively) and electron micrographs showing ultrastructural features of their cambium cells (D-I).

Reference

Tognetti, V. B.; Palatnik, J. F.; Fillat, M. F.; Melzer, M.; Hajirezaei. M.; Valle. E. M.; Carrillo, N. Functional replacement of ferredoxin by a cyanobacterial flavodoxin in Tobacco confers broad-range stress tolerance. *The Plant Cell* **2006**, 18, 2035-2050.
